# Supplementary figures and images for: Preserving cultural heritage: Analyzing the antifungal potential of ionic liquids tested in paper restoration
Source: PLoS One. 2019 Sep 17;14(9):e0219650. doi: 10.1371/journal.pone.0219650 (PMC6748409; doi:10.1371/journal.pone.0219650)

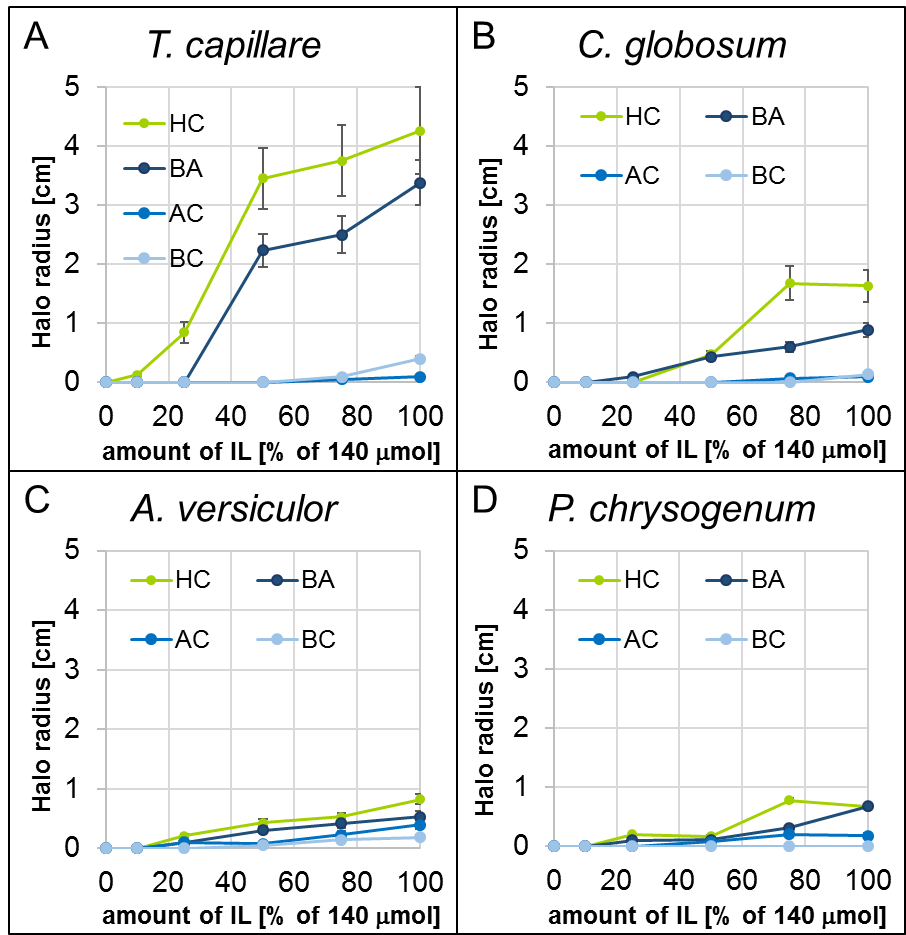

Supplement: S1 Fig — As seen for P. glabrum, HC had the strongest effect on all tested fungi, followed by BA, AC and BC. T. capillare (a) did show the strongest susceptibility to the tested ILs, followed by C. globosum (b), P. glabrum, A. versicolor (c) and P. chrysogenum (d). (TIF) [file pone.0219650.s001.tif]

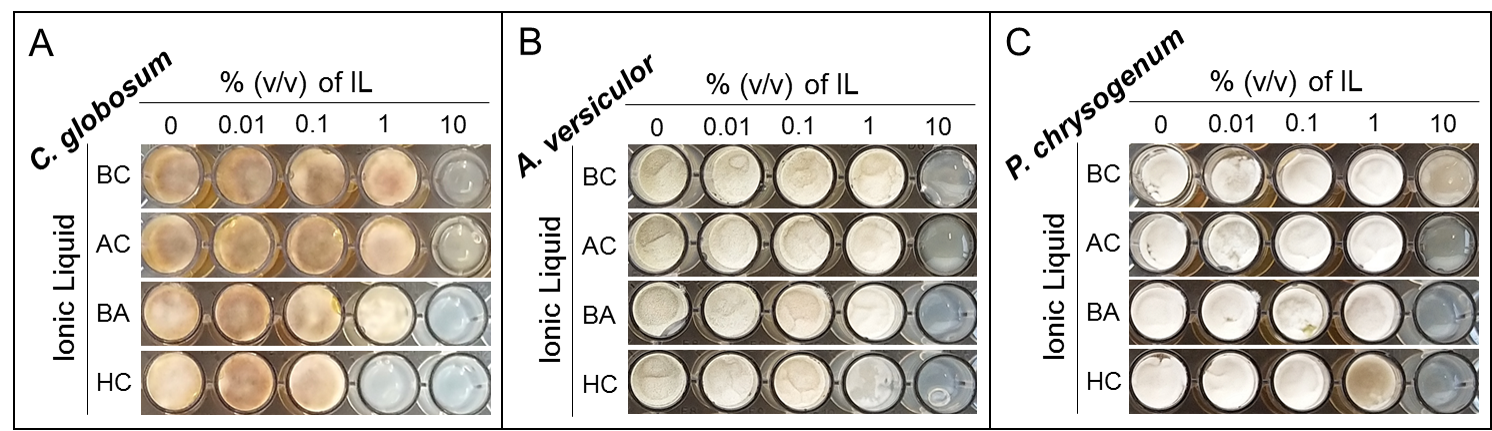

Supplement: S2 Fig — HC inhibited growth of C. globosum at 1% (v/v) and impaired growth of A. versicolor and P. chrysogenum at this concentration. BA was further shown to mildly impair growth of C. globosum at 1% (v/v). All ILs but BC were able to inhibit growth of all tested fungi at 10% concentration, stringently. (TIF) [file pone.0219650.s002.tif]
