# Supplementary material for: Preserving cultural heritage: Analyzing the antifungal potential of ionic liquids tested in paper restoration
Source: PLoS One. 2019 Sep 17;14(9):e0219650. doi: 10.1371/journal.pone.0219650 (PMC6748409; doi:10.1371/journal.pone.0219650)
Supplement: Attachment — Submitted filename: Response To Reviewers.docx [file pone.0219650.s003.docx]

**Response to the reviewers**

Dear reviewers,

We would like to gratefully thank you for your kind appreciation of our work and for your critical review of our article "Preserving cultural heritage: Analyzing the antifungal potential of ionic liquids tested in paper restoration". We value your constructive remarks and would like to respond to them point-by-point in the following:

**Reviewer #1 (D. S.):**

The manuscript presented by Schmitz entitled "*Preserving cultural heritage: Analyzing the antifungal potential of ionic liquids tested in paper restoration*" can be accepted after clarification of the following points:

1. The experiments are performed taking DMSO as the medium which itself shows zwitterionic properties and bear a appreciable value of polarity. So in order to understand the effects of the planted Ionic liquids, other solvent mediums like ethanol, acetone, ethers may be done in presence of ionic liquids. Also blank tests in these solvents have to be done and reported with comparisons with DMSO.

*We highly appreciate your constructive suggestions. However, as the original process, in which our ILs are applied, involves initial solubilization and subsequent precipitation of cellulose onto printed document surfaces, we are tightly limited in terms of the solvents we can use. Ethanol and acetone, for example, do not facilitate solubilization of cellulose fibers and strongly impair legibility of the treated documents due solubilization of the ink. We would also like to clarify that DMSO was only used as a solvent for Clotrimazole and 1-Butyl-3-Methylimidazolium chloride (both solid at room temperature), while the other ionic liquids were applied as pure liquids (without the need for additional solvents during potency assessment in this study). In order to clarify the methodological sequence for the reader, we made some amendments in the methods section. Moreover, a blank test of the antifungal activity of DMSO had already been included in the initial antifungal activity screening (Figure 3 A, B). Overall, we therefore do not think that additional tests with further solvents would give relevant results that could actually be used in the process.*

2. The authors have presented a model of the spatial orientation of the ionic liquid, solvent on cellular fibers on paper in Fig.2. The logical design may be explained clearly.

*We believe that the reviewer is actually referring to Figure 1 and – after reconsideration - agree with the reviewer that the initial illustration was partially misleading in terms of how the ionic liquid and DMSO entities were positioned. We have therefore now adapted the figure including its figure legend accordingly. We believe that the figure is now more accurately representing the process.*

**Reviewer #2 (R. S.):**

Paper is good and very much applied aspects. It definitely open a new area of research in the field of conservation work. But, personally, I feel if there is scope for modification, especially in discussion section.

*We have gladly modified the discussion section and hope to have improved it by doing so. Firstly, we have re-arranged the discussion paragraphs to better mirror the presentation sequence of the results. We believe that this allows a better orientation for the reader and hence could benefit the level of comprehensiveness.*

*We have rearranged the last bit of the first paragraph (i) to bring the sections discussing the correlation of IL potency and IL structure closer together in the text and (ii) to be able to discuss the long-term stability of the antifungal activity in the broader context of the paper-based application.*

*We furthermore included a statement in the second paragraph that points out the necessity for further studies on the contribution of non-polar group size and structure to the respective antifungal activity of an ionic liquid – as was done in the third paragraph as well.*

*Lastly, we extended the second-to-last paragraph. We aimed at putting stronger emphasis on the proposed future directions for research on biotic preservation implementation in paper restoration processes.*

*Additional minor modifications were made to improve overall readability and to clarify certain statements.*
